# The growth and development of children born to adolescent mothers in Taiwan

**DOI:** 10.1186/s13052-016-0280-5

**Published:** 2016-08-31

**Authors:** Wei-Ya Wu, Chi-Rong Li, Ching-Pyng Kuo, Yi-Chen Chiang, Meng-Chih Lee

**Affiliations:** 1 Institute of Medicine, Chung Shan Medical University, Taichung, Taiwan; 2 Public Health Center of Wurih District, Taichung, Taiwan; 3 School of Nursing, Chung Shan Medical University, Taichung, Taiwan; 4 School of Public Health, Chung Shan Medical University, Taichung, Taiwan; 5 Department of Family Medicine, Ministry of Health and Welfare, Taichung Hospital, 199, Sec. 1, San-Min Road, Taichung, Taiwan

**Keywords:** Growth, Development, Children, Adolescent mother, Taiwan Birth Cohort Study

## Abstract

**Background:**

Adolescent pregnancy carries a higher risk of adverse birth outcomes. Currently, there are very few longitudinal studies that have investigated the growth of children born to adolescents. This study explores the birth outcomes and determinants in adolescent pregnancies with subjects enrolled from the Taiwan Birth Cohort Study (TBCS).

**Methods:**

Using the data of Wave I (6 months old), II (18 months old), and III (36 months old) of TBCS, a national sample of 19,381 pairs of mothers and their children were included for analysis. Out of these subjects, therewere560 pairs of adolescent mothers and children. Through completed field interviews with structured questionnaires, surveys with mothers or other family members, and with references to each child's birth certificate and Passport of Well-baby Care, the differences in birth outcomes, personal, pregnancy, and social profiles of the mothers were analyzed.

**Results:**

A total of 560 adolescent mothers (<20 years old) and 18,821 adult mothers (20–34 years old) were included in this study. There was no significant difference between the two groups in terms of parameters of children growth and development. The numbers (proportions) of failure in milestones at 3 years old in gross motor functions, fine motor function, language, and social/personal development of children born to adolescent mothers are 13(2.32), 34(6.07), 10(1.79), and 24(4.29 %), respectively; while there are 392(2.08), 1015(5.39), 308(1.64) and 512(2.72 %) for those born to adult mothers, respectively. The risk factors of failure in children development were identified as “the mother isn't the night-time caregiver” and “family dysfunction”.

**Conclusion:**

There was no significant difference in development at 3 years old among children born to adolescent and adult mothers.

## Introduction

Adolescent pregnancy carries a higher risk of adverse birth outcomes. Currently, there are very few longitudinal studies that have investigated the growth and development of children born to adolescents. This study explores the birth outcomes and determinants in adolescent pregnancies, using subjects drawn from the Taiwan Birth Cohort Study (TBCS).

## Background

Children born to adolescent mothers have higher rate of premature birth, low birth weight, and other adverse birth outcomes [1]. However, currently there are very few longitudinal studies that have investigated the growth and development of children born to adolescent mothers after birth. In our previous investigation [2], it was determined that the average weight of children born to adolescent mothers, at 18 months-old, is no different than those born to adult mothers; and the average height is also approaching the same values. This result indicates that the development of children born to adolescent mothers has a steeper slope when compared to those born to adult mothers [3, 4]. The significant factors in affecting the children’s growth and development include: breastfeeding” and “adolescent mother is the primary daytime caregiver”. In order to understand the long term growth and development of these children, from birth to 3 years of age, a longitudinal follow-up study is required to determine the significant predicting factors for development. The TBCS serves as an important database for investigating the growth and development of children born to adolescent mothers. The result of this study can serves as a reference for future child care and health care education for expecting parents.

## Methods

### Design

The study was conducted using the data from a population-based TBCS that included a total of 24,200 pairs of mothers and newborns in 2005. This TBCS provides a wide range of longitudinal information regarding the children’s development. Infants with major disorders and congenital birth defects were excluded from this study. The study was reviewed and approved by the Medical Ethics Committee and Data Protection Board in Taiwan prior to commencement. After obtaining written informed consents, all eligible subjects (mother-child dyads) were categorized into two groups according their maternal ages at the time of delivery. The consent study subjects included878 adolescent mothers (≤20 years at the time of the child’s birth) and 20,370 adult mothers (aged 20 or older).

### Data collection

Data was collected by trained research assistants with the use of a structured questionnaire given during home interviews. The first and secondary home interviews with the 24,200 post-partum women were conducted at birth, 6, 18 and 36 months after their deliveries, during the period from June 2005 to December 2008. A total of 19,910 women completed the interviews, where the response rate was 82.27 %. Aside from mother and infant related information, family related information were also collected using the APGAR scale that was developed by Smilkstein et al., where scores from 0 to 7 were determined as family dysfunction. Information related to birth characteristics, including birth weight, birth order, gender, gestational age, head circumference, birth number (singleton or multiple pregnancy), method of delivery, patterns of infant feeding, and caregiving status were obtained. All of the weight, and length or height measurements were retrieved from the “Child Health Handbook”, which is the documented record of periodic health examinations (at the 1, 6, 18, 24, 30, and 36^th^ month) for all children up to 6 years of age (a free-of-charge service provided by the National Health Insurance Program in Taiwan). The weights and lengths or heights measurements were performed by trained nurses and recorded to the nearest 100 g and millimeter (mm), respectively. Feeding practice was described by a categorical variable and included as a time-dependent variable to assess its association with growth rate.

In terms of infant development, the TBCS utilized a Taiwan Infant Development Index that is based on the Denver Development Screening Test (DDST) [5]. The categories in the index include gross motor functions, fine motor functions, language, and social/personal developments. The inability to perform certain predefined tasks was considered as incompletion of a category.

Development Completion Definitions: Completed by a 3-year-old infant

- Gross motor functions:—“Can walk on their own without falling”
- Fine motor function:—“Can use pen-like objects for drawing random patterns”
- Language:—“Can imitate adult conversations”
- Social/personal:—“Can imitate actions performed by others”

### Analysis

Statistical analysis was performed using Statistical Analysis System (SAS) version 9.2 for Windows (SAS Institute Inc. Cary, NC, USA). Categorical variables are presented as count and percentage, where further comparisons were performed with chi-squared test or Fisher's exact test. Continuous variables are summarized by mean and standard deviation, and compared with a two-tailed independent sample *t*-test reported at a 0.05 significance level. Means, medians, standard deviations, frequencies, and percentages were calculated for descriptive data. Trend and differences in growth for body weight, body height, and head circumference among babies born to adolescent and adult mothers were analyzed using repeated measure analysis of variance (ANOVA) and Z test statistics, which is comparable across subject ages and provides a more sensitive assessment of deviations of growth. The variables of growth and the change of growth with time from birth to 18 months old were analyzed through a generalized estimating equation (GEE) modeling approach. The definition was used for logistic regression analysis to evaluate the association among growth and each of the dependent variables.

## Results

According to the TBCS database, in 2005, 4.13 % of newborns were born to adolescent mothers (Table 1). There was no significant difference in the male/female ratio of infants born to adolescent and adult mothers. However; the frequency of boys was 5 % higher in the adult mothers’ group. There were a higher percentage of adolescent mothers who gave birth via regular delivery than adult mothers (81.0 % vs. 65 %, *P* < 0.001). The major statistical significant demographic differences between adolescent and adult mothers were: primary night-time caregiver (adolescent mothers vs. adult mothers; 69.82 % vs. 49.24 %) and family dysfunctions (adolescent mothers vs. adult mothers; 46.79 % vs. 40.32 %). Furthermore, 64.7 % of adolescent mothers have been the caregivers of their infants up to 18 months, which was statistically significantly higher than the adult mothers.

**Table 1:** Demography of mothers and their children

Parameters at 6 months of age	Adolescent mothers		Adult mothers		*P* value
	*N*	%	*N*	%	
Gender					
Male	288	51.43 %	10150	53.93 %	.506
Female	272	48.57 %	9158	48.66 %	–
Postpartum depression					
Yes	84	15.00 %	3383	17.97 %	.983
No	471	84.11 %	15904	84.50 %	–
Primary night-time caregiver					
Not mother	165	29.46 %	9962	52.93 %	.00094
Mother	391	69.82 %	9267	49.24 %	–
Birth weight					
<2500 g	54	9.64 %	1297	6.89 %	.051
≧2500 g	505	90.18 %	17969	95.47 %	0–
Premature birth					
<37 weeks	80	14.29 %	2137	11.35 %	.691
≧37 weeks	480	85.71 %	17171	91.23 %	–
Family function					
dysfunction	262	46.79 %	7589	40.32 %	.013
Normal	284	50.71 %	11590	61.58 %	–
Total	560	–	18821	–	–

**Fig. 1:**
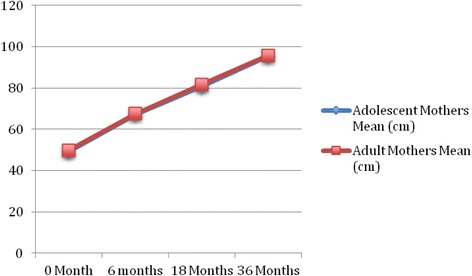
Trend and changes in body height among the children

**Fig. 2:**
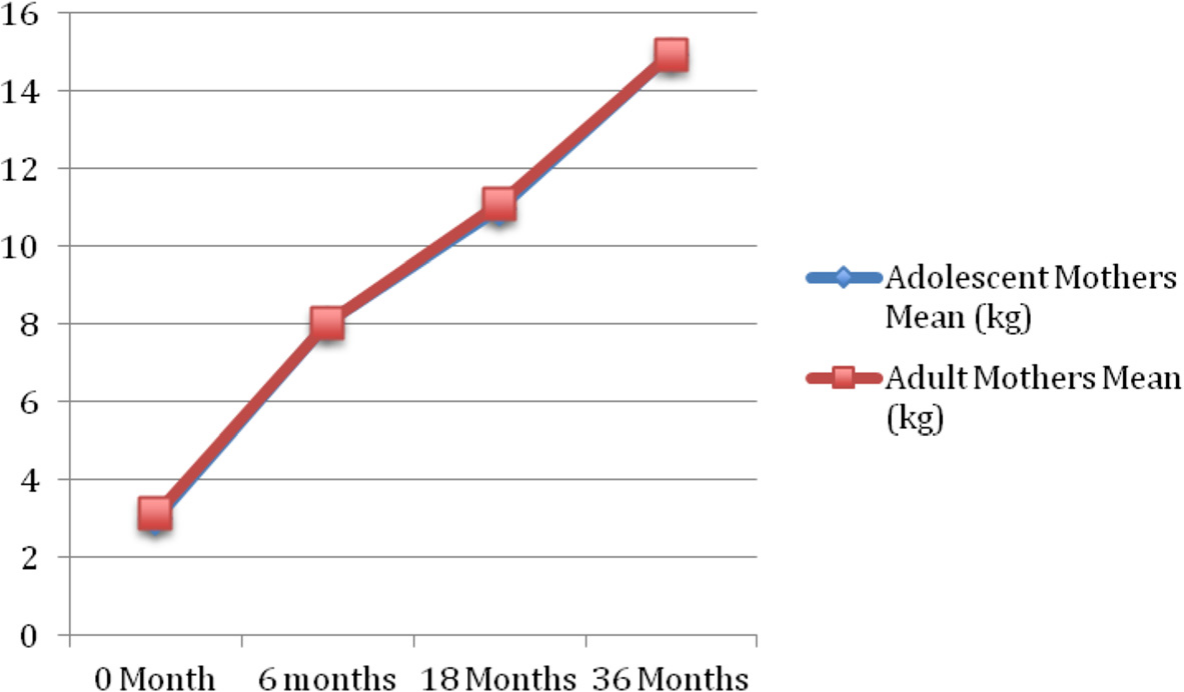
Trend and changes in body weight among the children

The growth patterns of infants born to adolescent and adult mothers are depicted in Figs. 1 and 2; indicating that the speed and slope of growth were similar in terms of body weight (BW) and body height (BH),from birth to 36 months, between the two groups. However, there exist significant differences between the groups for each stage of infant development (Table 2). Trend analysis of infants between the two groups showed no significant change or gain in overall weight (Table 3). Although there was significant difference in changes from BH to 18 month old (*P* < 0.001), no difference was observed at36 months. Similarly, the difference in BW was only observed at birth, and was not different at 36 months (*P* < 0.001, Table 2). At 3 years of age, children born to adolescent mothers exhibited no difference to those born to adult mothers in terms of gross motor functions, fine motor functions, language, and social/personal developments. For each developmental milestone s, children born to adolescent mothers have higher incompletion rate of fine motor functions and social/personal developments, however, the differences are not statistically significant (Table 4). The logistic regression analysis determined that in terms of social/personal development, the significant risk factors for incompletion at 3 years of age are: boys, adolescent mothers, and non-mother as primary night-time caregivers; in terms of fine motor skills, the significant risk factors are: girls, preterm birth, low birth weight, mother with postpartum depression, mother is the primary night-time caregiver, and family dysfunction.

**Table 2:** Trend and changes in body height (length) growth amount children

	Adolescent mothers		Adult mothers		*p* value
	Mean (cm)	SD (%)	Mean (cm)	SD (%)	
0 Month	49.26	2.59	49.76	2.51	<0.001
6 months	67.22	2.83	67.56	2.79	.027
18 Months	80.81	3.39	81.71	3.32	<0.001
36 Months	95.54	3.82	95.87	4.14	.074

Statistical significant: *p* < 0.001

**Table 3:** Trend and changes in body weight (length) growth amount children

	Adolescent mothers		Adult mothers		*p* value
	Mean (kg)	SD (%)	Mean (kg)	SD (%)	
0 Month	3.00	0.46	3.13	0.43	<0.001
6 months	8.05	1.08	8.05	0.98	.943
18 Months	10.98	1.36	11.11	1.33	.109
36 Months	14.95	2.17	14.95	2.20	.969

Statistical significant: *p* < 0.001

**Table 4:** Rates of completion for milestones in children development at 36 months

	Adolescent mothers				Adult mothers				*p* value
	Complete		Incomplete		Complete		Incomplete		
Development	*n*	%	*n*	%	*n*	%	*n*	%	
Gross motor functions	547	97.68 %	13	2.32 %	18429	97.92 %	392	2.08 %	0.42
Fine motor function	526	93.93 %	34	6.07 %	17806	94.61 %	1015	5.39 %	0.42
Language	550	98.21 %	10	1.79 %	18513	98.36 %	308	1.64 %	0.42
Social/personal	536	95.71 %	24	4.29 %	18309	97.28 %	512	2.72 %	0.1

## Discussion

In this study, the population-representative TBCS database was used to examine the correlation among children bone to adolescent mother or adult mother and the associated growth developmental changes over the first 18 months of life. Children born to adolescent mothers had significantly higher rates of lower birth BW, BH, and then those born to adult mothers. During the following 36 months, no significant difference in the speed and slope in weight gain and height gain was observed between the groups. Furthermore, children of adolescent mothers exhibited no difference in BW at 18 months, however, the BH difference remained until 36 months (shown in Figs. 1 and 2) when compared to those born to adult mothers (Table 2). Since this study is a longitudinal follow-up of national representative sampled population, we can conclude that the delay in BW and BH development of children born to adolescent mothers, between 6 and 18 months, is improved at 36 months.

In terms of developmental milestones at 36 months, children born to adolescent mothers showed no difference in gross motor functions, fine motor function, language, and social/personal development than those born to adult mothers (Table 5). It is possible that 36 months is not a sufficient duration for any significant difference to development. Therefore, additional investigation will be performed to determine any long-term deviations. A previous study performed by our research team [6] indicated that the development of children younger and 36 months of age is affected by factors such as breastfeeding and incinerator in the vicinity of residence. However, this study is focused on the development at 3 years of age, thus the effect of breastfeeding was not investigated. The effect of primary night-time caregivers and family dysfunction in the fine motor functions and social/personal development of children should be noted when providing support and care for adolescent mothers. The assessment index used in this study is based on the DDST. We are currently developing a Taiwan Infant Development Index (Barring), with support from the TBCS, for establishing an assessment that is more akin to the infant development environment in Taiwan. This population-based cohort study has several strengths [7]. The database provides a large national sample, thus the findings can be represented nationally [8]. The longitudinal nature of the data, with information provided over a long period of time, is better for assessing causal relationships than cross-sectional designs. There were also some limitations of this study that must be addressed. In this paper, physiological measurements were analyzed. However, environmental exposures (e.g. smoking, iodine deficiency, and alcohol intake) and genetic variants associated with several different outcomes were not evaluated. Some potentially relevant factors such as maternal pre-pregnancy body mass index, gestational weight gain, and gestational diabetes may be related to child growth and development, yet were not available. Apart from breastfeeding, insufficient data were collected for complementary feeding. Therefore, we are unable to address potential nutritional explanations for the apparent growth changes that were observed toward the end of the child’s third year of life.

**Table 5:** Logistic regression for milestones completion

Infant development milestones				
	Parameters	β	SE	OR (95 % CI)
Gross motor functions				
	Gender (female vs. male)	0.09	0.04	0.09 (0.01 ~ 0.18)
	Maternal perceived health status	3.05E–04	1.38E–04	3.05E–04 (3.3754E-05 ~ 5.76E-1)
Fine motor function				
	Gender (female vs. male)	0.12	0.07	0.12 (0.01 ~ 0.25)
	Postpartum Depression (no vs. yes)	0.12	0.04	0.12 (0.03 ~ 0.20)
	Night-Time Caregiver (mother vs non-mother)	0.19	0.05	0.19 (0.09 ~ 0.28)
	Low Birth Weight (yes vs. no)	0.25	0.09	0.25 (0.09 ~ 0.42)
	Family (dysfunction vs. normal)	0.18	0.03	0.18 (0.12 ~ 0.25)
	Preterm Labor (yes vs. no)	0.42	0.07	0.42 (0.29 ~ 0.56)
	Maternal perceived health status	1.98E–04	3.23E–04	1.98E–04 (4.33E-3 ~ 8.30E-3)
Language				
	Gender (female vs. male)	0.71	0.06	0.71 (0.59 ~ 0.84)
	Low Birth Weight (yes vs. no)	0.4	0.16	0.4 (0.08 ~ 0.72)
	Family (dysfunction vs. normal)	0.22	0.06	0.22 (0.10 ~ 0.34)
	Preterm Labor (yes vs. no)	0.25	0.13	0.25 (0.01 ~ 0.51)
Social/Personal				
	Low Birth Weight (yes vs. no)	0.25	0.18	0.25 (0.10 ~ 0.60)
	Maternal age	0.28	0.14	0.28 (0.00 ~ 0.55)
	Gender (female vs. male)	0.27	0.05	0.27 (0.17 ~ 0.38)
	Night-Time Caregiver (mother vs non-mother)	0.17	0.08	0.17 (0.01 ~ 0.33)

## Conclusions

Teen pregnancy and parenting remain important public health issues that deserve continued attention. It is more difficult for adolescent mothers to engage in a mature and sensitive manner with their children as a consequence of the presence of more risk factors. All of these issues can potentially affect the outcomes of the infants of adolescent mothers. The first year postpartum is a particular challenging period for adolescent mothers, as they cope with distinctive personal and social changes. Although children born to adolescent mothers were determined to be not at much greater risk for growth delays than their peers born to adult mothers in our study, their developmental outcomes at older ages remain unknown. This is because we could only characterize infant growth changes in weight and length in the predefined period of time like the other studies. There is a fundamental need for promoting adolescent mothers’ mental and physical health, and care giving abilities for providing proper care for their children during the early years. The results from this study indicated: the role of the primary night-time caregiver can affect children development especially in fine motor functions and social/personal development [9]. Therefore, we encourage adolescent mothers to return to their studies or professions, but are best to still provide night-time care for their children when they are at 3 years of age.

Additionally, the mother’s physiological status was shown to impact the child's development (i.e. postpartum depression). Therefore, more maternal detailed information should be investigated in future studies. Therefore, the development of postpartum depression must be especially noted. Finally, we look forward to this continual investigation as the focus of the TBCS shifts toward children development (in terms of physical, temperamental, and characteristic) beyond the age of 3.

There is no observable different among children born to adolescent and adult mothers at 3 years of age. Further study will be performed to understand future developmental changes and differences. We conclude that there is no significant difference in developmental characteristics of children born to adolescent mothers at 3 years of age, thus a further long-term investigation is warranted. Finally, additional factors such as father and other family information will be investigated.

## Abbreviations

ANOVA, analysis of variance; BW, body weight; BH, body height; DDST, Denver Development Screening Test; GEE, generalized estimating equation; mm, millimeter; TBCS, Taiwan Birth Cohort Study; SAS, Statistical Analysis System
